# ACI-GNN: Lightweight All-Channel Interaction Graph Neural Network for Multi-Sensor Coal-Rock Cutting Recognition

**DOI:** 10.3390/s25226820

**Published:** 2025-11-07

**Authors:** Zhixin Jin, Jie Cheng, Wenyan Cao, Hongwei Wang, Jiaxin Zhang, Zeping Liu, Haoran Wang, Jianzhong Li

**Affiliations:** 1 College of Safety and Emergency Management Engineering, Taiyuan University of Technology, Taiyuan 030024, China; jinzhixin@tyut.edu.cn (Z.J.); 18979079502@163.com (J.C.); wanghongwei01@tyut.edu.cn (H.W.);; 2 Coal Mine Intelligent Equipment Research Center of Shanxi Province, Taiyuan University of Technology, Taiyuan 030024, China; zhangjiaxin0903@gmail.com; 3 Xinjiang Intelligent Equipment Research Institute, Aksu 843000, China; 4 College of Mining Engineering, Taiyuan University of Technology, Taiyuan 030024, China; 5 State Key Laboratory of Intelligent Mining Equipment Technology, Taiyuan 030032, China; liuzeping517@163.com (Z.L.);; 6 Shanxi TZCO Intelligent Mining Equipment Technology Co., Ltd., Taiyuan 030032, China

**Keywords:** convolutional neural network (CNN), coal-rock recognition (CRR), all-channel interaction (ACI), multi-sensor data fusion

## Abstract

To address the current challenges of low single-sensor recognition accuracy for coal and rock cutting states, redundant channel feature responses, and poor performance in traditional neural network models, this paper proposes a new multi-sensor coal and rock cutting state recognition model based on a graph neural network (GNN). This model, consisting of a feature encoder, an information exchange module, and a feature decoder, enhances the communication of feature responses between filters within the same layer, thereby improving feature capture and reducing channel redundancy. Comparative, ablation, and noise-resistance experiments on multi-sensor datasets validate the effectiveness, versatility, and robustness of the proposed model. Experimental results show that compared to the baseline models, CNN3, ResNet, and DenseNet achieve improvements of 2.47%, 2.78%, and 1.50%, respectively. With the addition of the ACI block, the ResNet model achieves the best noise-resistance performance, achieving an accuracy of 93.27% even in 6 dB noise, demonstrating excellent robustness. Embedded deployment experiments further confirmed that the proposed model maintains an inference time of less than 216.1 ms/window on the NVIDIA Jetson Nano, meeting the real-time requirements of actual industrial scenarios and demonstrating its broad application prospects in resource-constrained underground working environments.

## 1. Introduction

The intelligence of coal mining, especially the intelligence the tunneling faces, is still in its early stages [1]. In this field, recognizing the coal-rock cutting state as a frontier technology for the adaptive cutting of tunneling machines increasingly highlights its urgency [2]. By real-time sensing of the coal-rock cutting state, the efficiency of tunnel excavation can be improved, the formation quality of the tunnel cross-section can be ensured, and the wear of the cutting head caused by load mutations can be effectively reduced [3]. Especially in high gas and coal dust environments, this technology also helps to avoid sparks generated by high-speed rock cutting, thereby reducing explosion risks. However, due to harsh conditions such as low light, noise, and water mist underground, the tunneling face currently relies mainly on the on-site personnel’s experience to judge the cutting state of the equipment. This seriously threatens the operators’ safety and hinders the unmanned mining process. To solve these problems and promote the development of this field, researchers have actively carried out a large amount of research work.

Benefiting from the rapid development of image and optical technology, researchers have begun to explore the direct use of machine vision to determine the cutting medium of the tunnel boring machine. Sun et al. [4] used a gray-level co-occurrence matrix for the feature selection of coal-rock images, achieving an average recognition rate of 94.12% in cross-validation. Si et al. [5] identified coal-rock laser point cloud data through an improved growth algorithm, achieving an accuracy of over 90%. Zhang et al. [6] used a two-dimensional discrete wavelet transform to compare the similarity of coal-rock images for recognition. Wang et al. [7] leveraged infrared thermal image characteristics for dynamic coal–rock interface identification, offering a solution resilient to the low-light conditions underground. Although these methods have made some progress in coal-rock recognition, image signals are often obstructed in actual working environments, and it is challenging to ensure real-time performance. Additionally, optical sensing equipment is expensive and requires high lighting conditions. Therefore, researchers use time series signals during the cutting process for recognition. Scholars such as Jiang et al. [8] established a theoretical model of mechanical signals and the proportion of coal and rock to complete recognition indirectly. Li et al. [9] proposed an improved adaptive noise complete ensemble empirical mode decomposition method to achieve coal-rock recognition based on audio signals. Many studies have proven the effectiveness of time series signals in coal-rock recognition, and it is generally believed that more types of sensors can improve the accuracy and robustness of the recognition task [10,11]. Based on this, Liu et al. [12] proposed a wavelet decomposition recognition method to extract features from vibration, pressure, and current signals. Wang et al. [13] established a feature database of coal-rock cutting current, vibration, and temperature signals, and used fuzzy D-S fusion theory for recognition. However, traditional signal processing methods require much domain-specific prior knowledge, and this complex and diverse feature engineering is not ideal [14,15,16]. Additionally, multi-sensor data fusion methods like the above often come with drawbacks such as data feature loss and high computational complexity, making them unsuitable for actual work environments with limited underground computing resources and model complexity constraints [17].

With the vigorous development of deep learning, Convolutional Neural Networks (CNNs) have accumulated many successful cases in time series signal classification tasks thanks to their advantage of automatically learning data features [18]. For instance, Huang et al. [19] proposed a multi-resolution self-supervised discriminant network, which effectively improved the performance of time series anomaly detection. Meanwhile, Zhang et al. [20] used a temporal convolutional network to achieve efficient compressed sensing and reconstruction of electromyographic signals. These works demonstrate the powerful ability of specialized deep architectures in capturing complex temporal dependencies and features. However, the conventional convolutional layers that form the backbone of these networks have inherent limitations. It is well known that conventional convolutional layers in neural networks consist of a set of filters with learnable weights and biases, which gradually respond to input data from lower to higher layers. However, these filters are essentially a set of neurons for three-dimensional input that can only observe local data and connect with spatially adjacent neurons through shared parameters [21]. Typically, information from different channels is extracted and processed independently, and if some channels capture similar features, they are still represented separately [22]. This design overlooks the interaction of response information between channels within the same layer, leading to redundancy in channel information. Many scholars have proposed channel attention mechanisms in the field of vision [23,24], but signal channel interaction in multi-sensor contexts has rarely been studied. In industrial time-series analysis, some researchers have begun to address inter-sensor relationships. Zhang et al. [25] proposed a multi-head dynamic graph attention network for fault diagnosis under speed fluctuations, explicitly modeling the dynamic dependencies between different sensor signals, which shares a similar motivation with our work to go beyond independent channel processing. In multi-sensor modes, the shortcomings of traditional models manifest as features potentially overly focusing on a specific channel of a particular sensor as the number of layers deepens, with a significant overlap of filter fields of view [26]. However, the ideal situation is that we hope the information from each channel can be widely transmitted, fully utilizing the information from different sensors or channels for effective learning.

To address this fundamental limitation of standard architectures in modeling inter-channel relationships, Graph Neural Networks (GNNs) [27] have emerged as a powerful paradigm for processing non-Euclidean data and modeling relationships between entities. Their ability to perform structured information interaction through message passing between nodes makes them particularly suitable for tasks requiring relational reasoning [28], such as capturing the complex dependencies between multiple sensor channels. This positions GNNs as a promising technological solution for the multi-sensor fusion challenge in coal-rock recognition.

The limitations of existing methods restrict the practical application of the current multi-sensor coal-rock cutting state recognition. Therefore, this paper proposes, for the first time, an all-channel interaction (ACI) module based on Graph Neural Networks (GNN). This plug-and-play module mainly consists of feature encoding, information interaction, and feature decoding. In the first part, the input features are mapped to a vector through a multilayer perceptron, achieving comprehensive perception and compression of signal features. The second part uses GNN to ensure information interaction between different channels, perceiving the correlations and contextual information between channels, thus enhancing the network’s expressive ability and performance. The third part is responsible for decoding the response features and correcting the weights according to the shape of the original input. Overall, compared to ordinary convolutional layers, this module is closer to how the human brain processes information. Ordinary convolutional layers typically process features locally and independently at each position, with relatively limited information interaction between channels. Correspondingly, neurons in the human brain form complex neural networks through connections, allowing extensive communication and sharing of information within the network [29].

**Figure 1:**
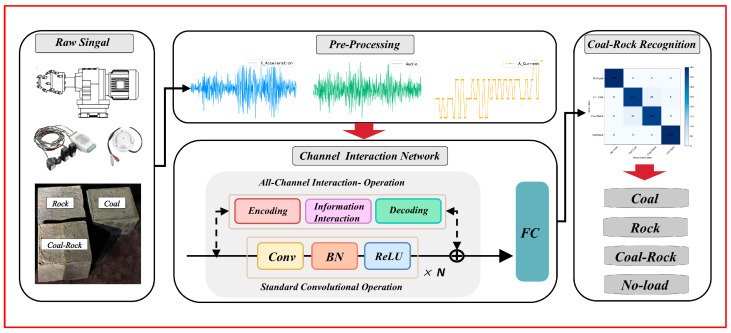
The framework for CRR using an all-channel interactive graph neural network.

This paper used GNN to study a multi-sensor coal-rock recognition method based on an all-channel interaction network. The overall flowchart is shown in Figure 1. The main contributions of this paper are summarized as follows:

(1) An experimental platform was constructed to recognize the coal-rock cutting state, collecting multi-sensor datasets under varying cutting conditions. These datasets included current, vibration, and audio signals. An adaptive sampling algorithm was proposed to preprocess the data, ensuring channel equalization while directly constructing input signals that are ready for analysis.
(2) Based on Graph Neural Networks, an ACI block was proposed, which reduces the accumulation of redundant information in channels, improves the filter’s ability to capture input features from different sensors, and ameliorates the channel overfocusing defects in multi-sensor scenarios.
(3) The ACI block can be seamlessly integrated into most mainstream model architectures, enhancing network performance without increasing computational load or memory usage. Ablation experiments were conducted to determine the optimal placement of the modules within different architectures.

The trained model was deployed on the NVIDIA Jetson Nano embedded platform, where experiments demonstrated the model’s real-time performance and potential for industrial applications.

## 2. Methodology

### 2.1. Formulation

**Figure 2:**
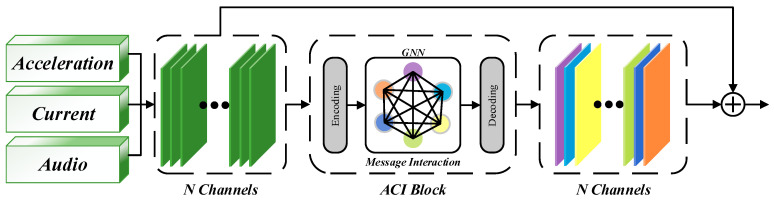
Overview of ACI block.

In Section 2, we will further introduce the ACI block, and the detailed structure of the module is shown in Figure 2. After preprocessing the signals from multiple sensors and concatenating them into input features, a sliding window is typically used to segment the input signals for easier recognition of the current cutting state. In actual work scenarios, each sliding window corresponds to a specific cutting state at a particular moment. When using the ACI block for state classification, assume that the current model structure contains *l* layers, and each layer’s neural network has $n_{l}$ units. For ease of presentation, let $X_{l}=\{x_{l}^{1},\;\ldots ,\;x_{l}^{n_{l}}\}$, where $X_{l}$ represents the feature responses of the *l*th layer, and define the input size height as *h* and width as *w*. After the features pass through the ACI block, the feature response is expressed by Equation (1):

(1) $${\hat{x}}_{l}^{i}=x_{l}^{i}+f_{l}^{i}(x_{l}^{1},\;\ldots ,\;x_{l}^{n_{l}}),$$

where the feature responses of all filters are accumulated and calculated through the function $f_{l}^{i}$, but in the calculation, only the response of the i-th channel is updated each time. This interaction mechanism with all filter feature responses is defined as an All-Channel Interaction. The Squeeze-and-Excitation block is widely used in various time series recognition models. Unlike the traditional SE block that uses a multilayer perceptron to implement the attention mechanism, we use the response function $f_{l}^{i}$, to make channels as nodes of the graph structure of the ACI block using a graph neural network, thus realizing the full range of information interaction between individual channels. We will detail the specific structure of the ACI block in Section 2.2.

### 2.2. Architecture

The All-Channel Interaction is mainly composed of three parts: the Feature Encoder, which compresses and perceives features through a multilayer perceptron; the Information interaction, which transfers response information across all channels via a Graph Neural Network; and the Feature Decoder, which decodes the updated response information into the shape of the input features.

(1) Feature encoding. The encoder processes sensor signal inputs by flattening the multi-channel feature map into a one-dimensional vector, preserving channel information, temporal correlation, and eliminating spatial correlation. Specifically, the multi-channel sensor data, initially organized as a matrix where each row represents a different channel and each column represents a measurement point, is transformed by concatenating the values from each channel into a single long vector. This vector is then passed through two fully connected layers, as represented by Equation (2).

(2) $$\begin{array}{c} y_{l}^{i}=f_{\mathrm{enc}}^{\mathrm{in}}(x_{l}^{i}),\\ z_{l}^{i}=f_{\mathrm{enc}}^{\mathrm{out}}(\sigma \left(y_{l}^{i}\right)). \end{array}$$

where $f_{enc}^{in}$ and $f_{enc}^{out}$ represent two linear functions, and σ represents the ReLU activation function, enabling the network to learn nonlinear relationships and better fit complex signal distribution. To reduce the network’s parameters and computational load, we designed a bottleneck after the function $f_{enc}^{in}$, compressing the input feature dimensions by a factor of γ > 1 while ensuring the effectiveness of the features.

**Figure 3:**
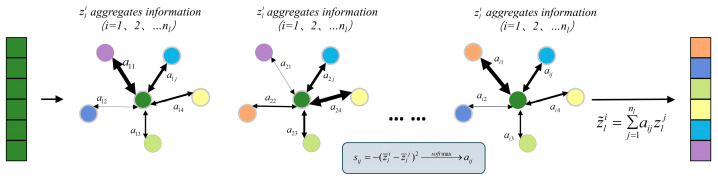
Channel information interaction: Arrow thickness indicates the attention weight (a_(ij)_), with thicker arrows representing higher feature similarity.

(2) Information interaction. To ensure that signals from multiple sensors contribute maximally to recognition, the Information interaction module transfers information across all channels and updates feature responses on time to better encode multi-sensor data features. Graph Neural Networks have become the mainstream framework for such channel interaction mechanisms [25,27]. Researchers combine GNN with the attention mechanism to form a soft attention mechanism [24,28], and the ACI block’s formula expression achieves a similar effect of channel interaction. In this paper, we construct a fully connected undirected graph in which each node corresponds to the feature response of a channel, thereby achieving direct information flow between all channels. The node set is defined as $Z=\{z_{l}^{i}\}$, and the edge strength between nodes as $s_{ij}=f_{str}(z_{l}^{i},z_{l}^{j})$ [28]. The edge strength represents the connection strength or relationship between channels, which directly determines the extent of information propagation in the graph. In recent years, researchers have proposed a large number of methods for calculating edge strength, this paper defines edge strength as represented by Equation (3):

(3) $$\begin{array}{c} {\bar{z}}_{l}^{i}=\sum_{k=1}^{h_{l}w_{l}}z_{l}^{i}[k]/(h_{l}w_{l}),\\ s_{ij}=-{\left({\bar{z}}_{l}^{i}-{\bar{z}}_{l}^{j}\right)}^{2}. \end{array}$$

where $z_{l}^{i}[k]$ represents the *k*-th element of the flattened long vector. By taking the expectation of the encoder’s output shape, the robustness of information interaction is increased. Meanwhile, we define an evaluation metric for the degree of channel interaction. By calculating the negative squared distance, channels with similar features interact more, increasing feature responses and enhancing recognition effectiveness. The distance calculation results are normalized through a softmax layer to obtain the attention weight $a_{ij}$. This process implements a graph attention mechanism. Each node aggregates information from other nodes based on the calculated attention weight *a_ij_* and finally updates its feature response. As shown in Figure 3. The final output set $\tilde{Z}=\{{\tilde{z}}_{l}^{i}\}$ is expressed as follows:

(4) $${\tilde{z}}_{l}^{i}=\sum_{j=1}^{n_{l}}a_{ij}z_{l}^{j},$$

(3) Feature Decoding. To facilitate residual calculations, the decoder reshapes the output set $\tilde{Z}$ to match the dimensions of the input features. The final decoder stage integrates the channel information with corresponding weights, producing ${\hat{x}}_{l}$ from Equation (1). This output is then forwarded to deeper layers for conventional convolution operations.

(4) Model Complexity. Considering the limited computational resources underground, the ACI block needs to improve recognition performance under minimal computational load. The computational increment of the ACI block only appears in 4 fully connected layers. Due to the compression effect of the bottleneck, we define the computational parameters of this layer as $N_{l}=\frac{4}{\gamma }\sum_{l=1}^{L}\left(H_{l}W_{l}\right)$2. This indicates that the parameter increment is independent of the number of channels. Subsequent experiments show that the ACI block does not need to be added after each convolution layer; it only needs to play its role in information interaction after certain key layers, further reducing the computational burden.

### 2.3. Response Assessment

The interrelationships between the features expressed by different channels in a specific layer can be understood by calculating the correlation between channel response maps. The correlation between features can help us understand whether the information encoded by different channels in a specific network layer is overlapping or diverse. Equation (5) for the correlation is expressed as follows:

(5) $$c_{l}^{ij}=\frac{\sum_{m=1}^{H}\sum_{n=1}^{W}(x_{l}^{i}[m,n]-{\bar{x}}_{l}^{i})(x_{l}^{j}[m,n]-{\bar{x}}_{l}^{j})}{\sigma_{x_{l}^{i}}\sigma_{x_{l}^{j}}},$$

where $\bar{x}$ and σ represent the mean and variance of the channel response $x_{l}^{i}$. If the correlation is high, indicating a large correlation value, it means that the feature expressions between different channels have significant overlap or redundancy, implying that the features learned by the network at this layer may lack diversity, and the information repetition rate is high. Conversely, if the correlation is low, indicating a small correlation value, it means that the feature expressions between different channels are more diverse. The features learned by the network at this layer are richer and more diverse, with a low information repetition rate, which is exactly what we need.

## 3. Experiments and Discussions

### 3.1. Experimental Platform Design and Data Description

#### 3.1.1. Data Acquisition

**Figure 4:**
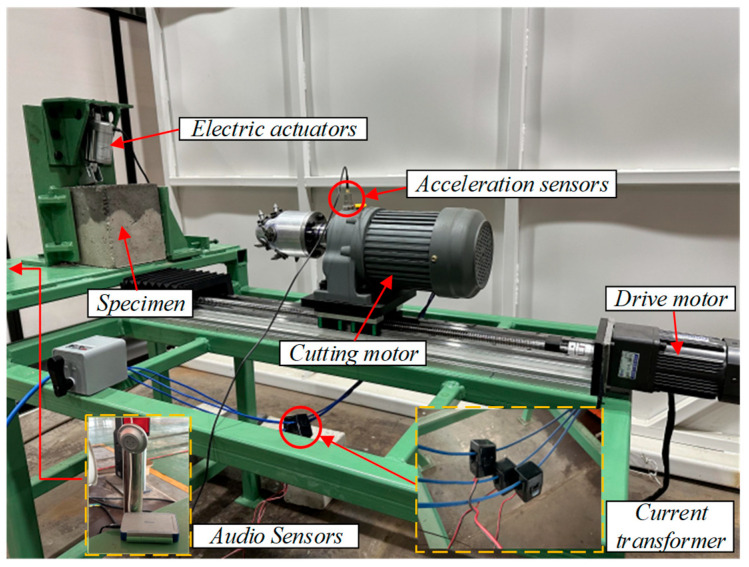
Experimental platform for coal-rock cutting and signal testing.

**Figure 5:**
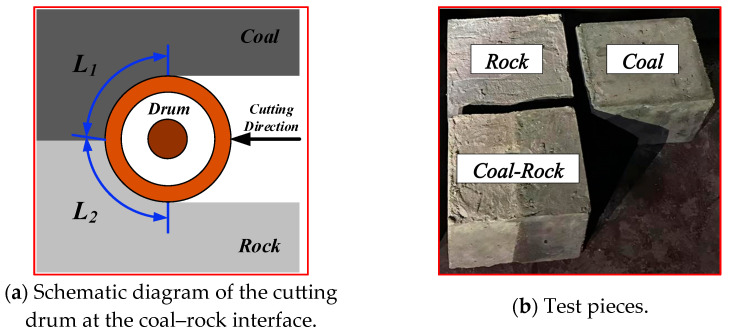
Test pieces in different proportions.

The experimental platform, designed based on similarity theory, uses the EBZ200 large road header as a model, including a cutting mechanism, a traveling mechanism, a fixed mechanism, and a signal measurement system, as shown in Figure 4. The cutting mechanism uses a 380 V three-phase asynchronous reduction motor (Wenling Xiushi Electrical Co., Ltd., Wenling, Zhejiang, China) with a rated power of 550 W and a rated speed of 1500 r/min. The reduction ratio can be adjusted through the gearbox. The cutting head’s maximum diameter is 120 mm, the effective cutting depth is between 50 mm and 70 mm, and the cutting speed is 67 r/min. The traveling mechanism consists of a lead screw slide and a 220 V variable speed motor with a rated power of 120 W, a reduction ratio of 1:15, and an output shaft speed range of 0–90 r/min. Forward and backward movement of the road header is simulated by controlling the forward and reverse rotation of the motor. The signal measurement system consists of a three-phase acceleration sensor CT1010 L (CT1010 L, Shanghai Chengke Electronics Co., Ltd., Shanghai, China), a three-phase current transformer DAM-3505 (DAM-3505, Beijing Altai Technology Development Co., Ltd., Beijing, China)., and an audio sensor, with signal acquisition frequencies of 800 Hz, 50 Hz, and 800 Hz, respectively. The specific parameters of the experimental platform are detailed in Table 1. Before collecting vibration data of coal-rock cutting, three different proportions of coal-rock specimens must be installed on the fixed structure. Top pressure is applied to the specimens through an electric push rod. Data collection starts as soon as the cutting head contacts the specimen surface. Each data sampling duration is no less than 10 s, and multiple repeated experiments are conducted for each sample to ensure data reliability. To simulate underground conditions, we used sand, granular coal, and PC32.5 composite Portland cement to prepare specimens, ensuring that the material properties of the coal and rock samples were similar to natural coal and rock. Three different proportions of coal-rock specimens were prepared in the experiment, representing the rock-cutting process (coal-rock ratio 0:1), the coal-cutting process (coal-rock ratio 1:0), and the coal-rock interface cutting process (coal-rock ratio 1:1), as shown in Figure 5a. The composition ratios of the specimens are detailed in Table 2 [13]. During the preparation of the specimens, we defined the contact arc lengths of the cutting head with coal and rock components, denoted as $L_{1}$ and $L_{2}$, respectively. The coal-rock ratio is determined by $L_{1}/L_{2}$. The prepared coal-rock specimens are shown in Figure 5b.

#### 3.1.2. Data Preprocessing

**Figure 6:**
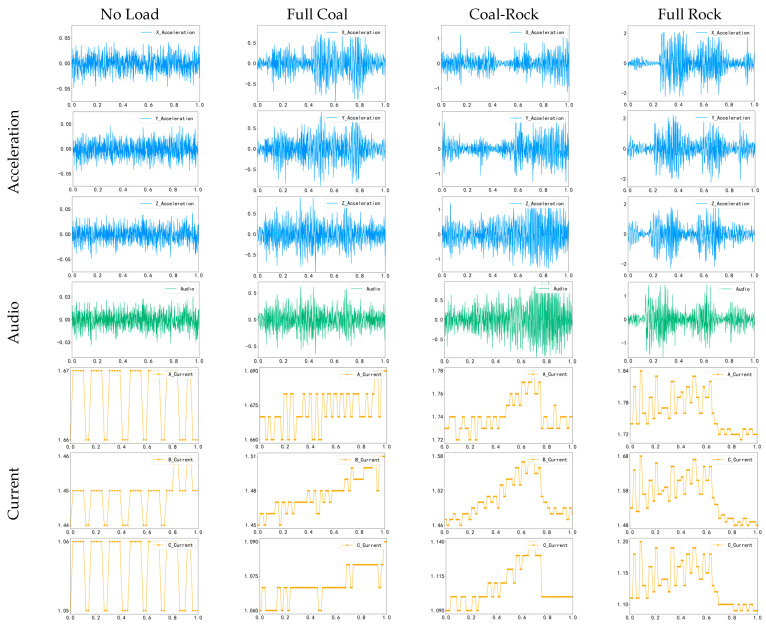
Vibration, audio, and current signals under four operating conditions.

The signals from the three sensors collected in the experiment are shown in Figure 6, including the X, Y, and *Z*-axis data from the acceleration sensor, audio data, and the A, B, and C pole data from the current sensor. Due to the differences in sampling rates among sensors, this study downsampled the acceleration and audio signals to 50 Hz. This choice was based on the system’s dynamic characteristics. The cutting head rotated at 67 rpm, corresponding to a fundamental frequency of approximately 1.1 Hz, indicating that significant vibration and acoustic signatures induced by coal cutting are primarily concentrated in the low-frequency region. According to the Nyquist sampling theorem, a 50 Hz sampling rate can represent signals up to 25 Hz, making it sufficient to capture essential dynamic characteristics relevant to identification, ensuring fairness in subsequent experiments and balancing data across channels.

This study employs a standard resampling algorithm to retain critical information and avoid noise or interpolation artifacts caused by simple time synchronization methods such as nearest neighbor interpolation. Our method, based on low-pass filtering followed by resampling, ensures smoother signal transitions without introducing irregular artifacts. The downsampling and concatenation algorithm mentioned above are shown in Algorithm 1. The final model input is a 9-channel signal consisting of the concatenation of the upscaled three-channel audio signal and the three-channel current signal, sampled at a rate of 50 Hz. The three-channel upscaling of the audio signal is primarily intended to ensure architectural consistency and enhance information representation. This ensures that the audio signal’s channel dimensionality is consistent with that of the acceleration and current signals, ensuring that the model can symmetrically and efficiently learn features from all sensors and avoiding structural biases for sensors with a large number of channels. To objectively evaluate the impact of this design choice, we conducted an ablation experiment, the results of which are detailed in Section 3.2.2.
**Algorithm 1:** Preprocessing algorithm: Synchronize and Concatenate Multi-Rate Multi-Channel Time Series Signals**Input:**    signal1 (N1, 3),    signal2 (N2, 3),    signal3 (N3, 1),    sr1, sr2, sr3**Output:** concatenatedSignal01. targetSR ← min(sr1, sr2, sr3)02. **Define** the resample function: 03.      **Function** Resample(signal, originalSR, targetSR): 04.          Apply low-pass filter to signal with cutoff at targetSR/2 05.          numSamplesTarget ← int(len(signal) × (targetSR/originalSR)) 06.          resampledSignal ← Resample(signal, numSamplesTarget) 07.          **return** resampledSignal 08. signal1 Resampled ← Resample(signal1, sr1, targetSR) 09. signal2Resampled ← Resample(signal2, sr2, targetSR) 10. signal3Resampled ← Resample(signal3, sr3, targetSR) 11. minLength ← min(len(signal1Resampled), len(signal2Resampled), len(signal3Resampled)) 12. signal1Aligned ← signal1Resampled[:minLength, :] 13. signal2Aligned ← signal2Resampled[:minLength, :] 14. signal3Aligned ← signal3Resampled[:minLength] 15. signal3Expanded ← Stack([signal3Aligned, signal3Aligned, signal3Aligned], axis = −1) 16. concatenatedSignal ← Concatenate([signal1Aligned, signal2Aligned, signal3Expanded], axis = −1) 17. **return** concatenatedSignal

There is no consensus on the optimal window size for deep learning. This paper refers to existing successful cases, segmenting the filtered signals into windows of 128 points in length and using these windows as units for segmented sampling [7,19,20]. The sampling duration is 2.56 s, which serves as the basic unit for segmented sampling. To better capture local features of the data, the window movement step is set to 50% of the window length (i.e., 64 points). After signal segmentation, the signal segments are classified according to four operating states, with class labels set as Class A (no load), Class B (full coal), Class C (coal-rock interface), and Class D (full rock), thereby constructing a complete dataset.

To evaluate model performance and ensure the reliability of the results, the dataset is divided into training and test sets in a 4:1 ratio. The partitioning process strictly uses a sliding window method, performed within the training and test sets, and ensuring that the windows do not overlap, effectively preventing overestimation of model performance due to data leakage. Ultimately, the test set contains 400 samples per class, for a total of 1600 test samples. The detailed composition of the dataset is shown in Table 3.

### 3.2. Discussion and Analysis of Experimental Results

For our experiments, we utilized the PyTorch 1.12.1 framework. The experimental environment was configured as follows: Operating System: Ubuntu 18.04; GPU: 2 × 16 GB Nvidia Tesla T4; CPU: Intel Core i7-10875H; RAM: 32 GB. We chose three commonly used neural network architectures: CNN3 (3 layers CNN) [30], ResNet [31], and DenseNet [32] as baseline models. Specifically, CNN3 consists of 3 convolutional layers, ResNet consists of 6 convolutional layers, and DenseNet consists of three dense blocks, each containing a convolutional layer, with transition layers connecting the dense blocks. The experiment used the Adam optimizer, set the learning rate to 0.0005, batch size to 128, Training epoch to 200 and CrossEntropyLoss was chosen as the loss function. The detailed model architecture and properties of each layer are shown in Table 4, where C(Ls) represents the number of feature maps in each convolutional layer. To verify the recognition effect of the ACI block on multi-sensor signals, we will insert the module into the above three mainstream models, demonstrating that the ACI block can achieve the recognition performance of deeper models with fewer parameters, improve the filter’s feature capture ability, and reduce channel redundancy. We also explore the optimal insertion position through ablation experiments, investigating the improvement in recognition effect due to encoding, decoding, and information interaction. Finally, to verify the robustness of the improved model, we conducted experiments by adding noise of different decibels to the test set. The design and methods of the experiments aim to comprehensively evaluate the effectiveness and performance improvement of the ACI block in multi-sensor signal recognition tasks, providing guidance and a basis for optimizing the model structure and parameter configuration.

#### 3.2.1. Comparative Experiments

Based on the original framework, we introduced an information interaction mechanism to enhance communication between channels. To compress features, we set α=8 in the experiment. We improved the performance of the optimal insertion position of the model, achieving the best average effect over five trials, as shown in Table 5 below. Compared to their respective baseline models, CNN3, ResNet, and DenseNet improved by 2.47%, 2.78%, and 1.50%, respectively. Comparison with existing attention mechanisms further demonstrates the superiority of the ACI module. It consistently outperforms the classic SE module across all architectures, achieving leads of 0.96%, 1.95%, and 1.06% on CNN3, ResNet, and DenseNet, respectively. Furthermore, our method comprehensively outperforms the newer CA module, with respective advantages of 0.53%, 1.22%, and 0.55%. These results fully demonstrate the effectiveness of our proposed full-communication approach in addressing channel interaction.

**Figure 7:**
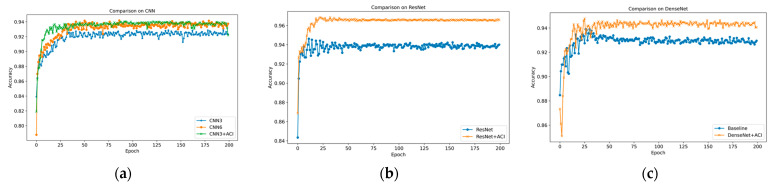
Dataset’s accuracy on different models. (**a**) CNN. (**b**) ResNet. (**c**) DenseNet.

**Figure 8:**
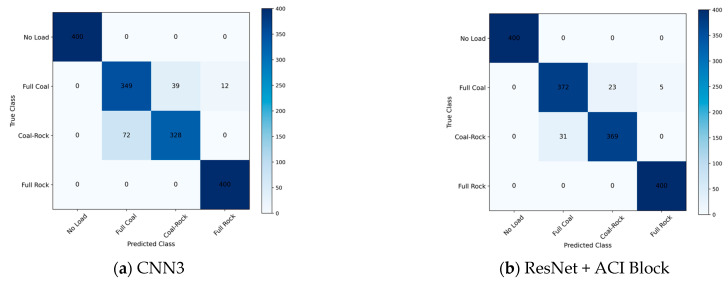
Confusion Matrix on different models.

Figure 7 shows the accuracy of the test set for the three model frameworks before and after adding the ACI block. Figure 8 calculates the confusion matrix on the dataset, where the diagonal indicates the number of correct identifications, and the darker the color, the higher the value. It is obvious that errors are more likely to occur when identifying the full-coal and coal-rock interface conditions, but the improved model significantly reduces this issue.

**Figure 9:**
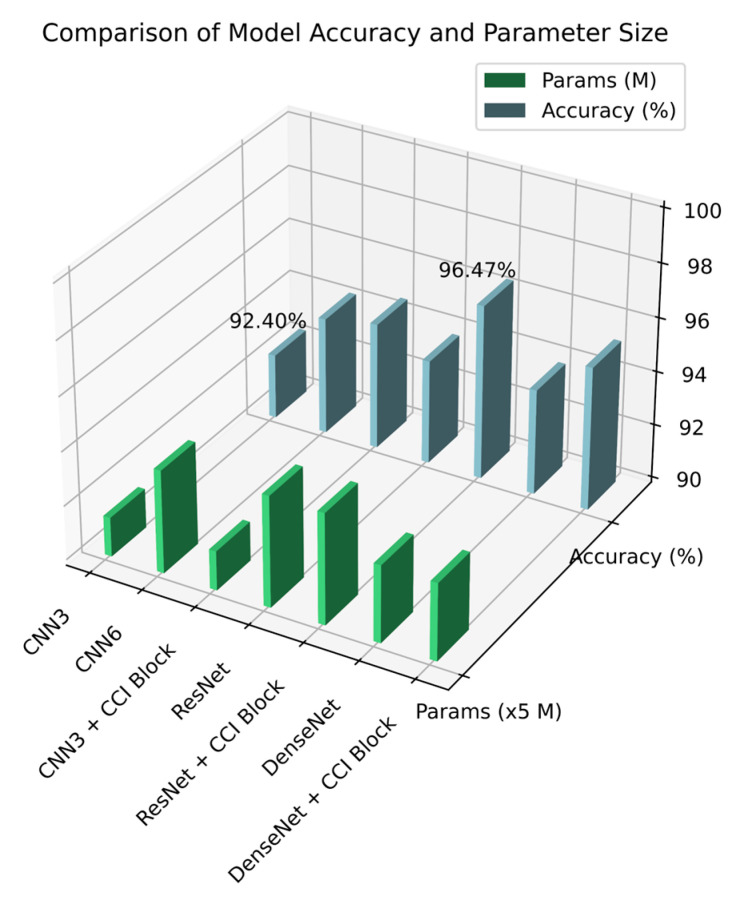
The classification accuracy and parameters for different models.

To prove that the ACI block can achieve the performance of deeper models with a small increase in computational parameters, we compared CNN3, CNN6 (6 layers CNN), and CNN3 + ACI block. The experimental results are shown in Figure 9. The results show that with almost equal performance, the model parameters were reduced by 62%. Therefore, we conclude that the ACI block can reduce the model’s depth effectively, achieving a good balance between model complexity and performance.

**Figure 10:**
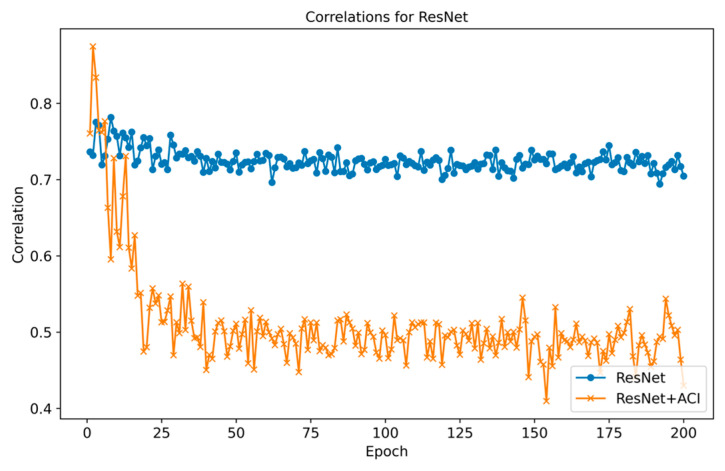
Correlations for ResNet at different epochs.

To directly verify the ACI module’s concept of reducing channel redundancy, we calculated the correlation between the feature responses of all channels in the key layer, both with and without the ACI module. The results are shown in Figure 10, which indicates that the correlation of the average feature responses of the channels is greatly reduced compared to the baseline, which verifies our conception of reducing channel redundancy and that the filters can capture more diversified features after the information interactions to improve the utilization of multi-sensor data.

#### 3.2.2. Ablation Experiments

(1) The optimal insertion position of the module

**Figure 11:**
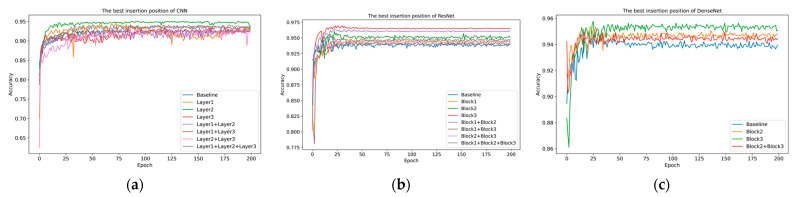
Accuracy of ACI block inserts in different positions. (**a**) CNN. (**b**) ResNet. (**c**) DenseNet.

Through ablation experiments on the multi-sensor dataset, we explored the optimal insertion position of the ACI block. We added the module in key positions as much as possible to reduce computational load rather than adding it between every convolutional layer. In the DenseNet structure, due to the significantly larger computation size after the first layer, we did not conduct insertion tests in this experiment. The experimental results are shown in Table 6, indicating that adding the ACI block after the second or third layer in the three model architectures significantly improves recognition performance. Figure 11 shows the accuracy of the test set. As the number of layers increases, the feature response size is reduced dramatically, and the computational burden for channel interaction is smaller than shallow insertion. This also aligns with recent research findings, where deeper layers extract higher-level semantics, allowing channels to obtain richer feature responses.

For the ResNet structure, adding the ACI block after residual calculation showed better performance. We believe this is because the original features before the residual calculation are retained, and the interaction with channels that learned new features will adjust the channel weights to more appropriate values. Similarly, the DenseNet structure’s unique feature reuse mechanism makes information interaction particularly important. As the dense blocks increase, the number of channels continues to grow, and inserting the ACI block in deeper positions can better adjust channel weights. DenseNet uses dense connections to take feature maps from all previous layers as input, and this feature reuse mechanism can also capture more diverse features at deeper levels, making the insertion of the ACI block more effective at these positions.

(2) The most important part of the module

**Figure 12:**
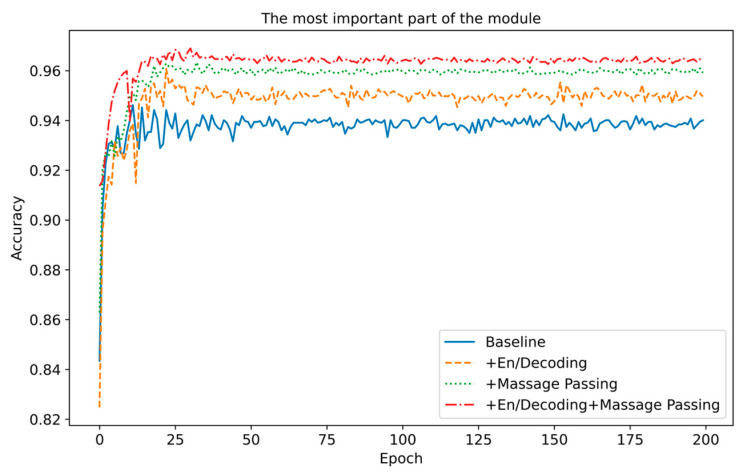
Comparison of accuracy on ResNet with different parts of the module.

To study the contribution of each component of the ACI block, we independently used the encoder/decoder and information interaction mechanism in the experiment. We chose the best-performing ResNet as the experimental model to simplify variable comparison. The experimental conditions and results are shown in Table 7 and Figure 12. The experimental results show that both parts have an optimization effect on the baseline model. The encoder/decoder, through the advantages of dimensional compression and feature capture, can enhance model performance to a certain extent; the Information interaction mechanism, through information interaction between channels, makes the filter feature responses more diverse, reduces channel redundancy, and thus achieves better results. When these two parts work together, not only is each channel globally perceived, but the network also learns the weight of each channel, significantly enhancing the model’s overall performance and achieving the optimal effect.

(3) Effectiveness Analysis of Audio Channel Extension.

To verify the rationality of audio channel expansion, while keeping all other hyperparameters, data preprocessing processes and model architecture unchanged, we selected the best-performing ResNet + ACI Block as the experimental model, and compared the following three input configurations: the baseline model proposed in this paper, which repeats the single-channel audio signal into three channels and then splices them together; the comparison model, which only processes the audio as a single channel and fills it with three channels by zero padding; and the audio-free model, which completely removes the audio data to evaluate its basic contribution. The specific results are shown in Table 8.

Experimental results show that expanding the audio signal channel does improve model performance, particularly in key metrics like accuracy and F1 score. Comparing this with removing audio data and zero-padding clearly demonstrates that expanding the audio channel does not unfairly bias model results. Instead, it provides a richer feature representation, helping the model better capture important information in the signal. Therefore, expanding the audio signal to three channels is a fair and effective design choice, and its performance gains stem from superior signal representation capabilities.

#### 3.2.3. Anti-Noise Experiment

**Figure 13:**
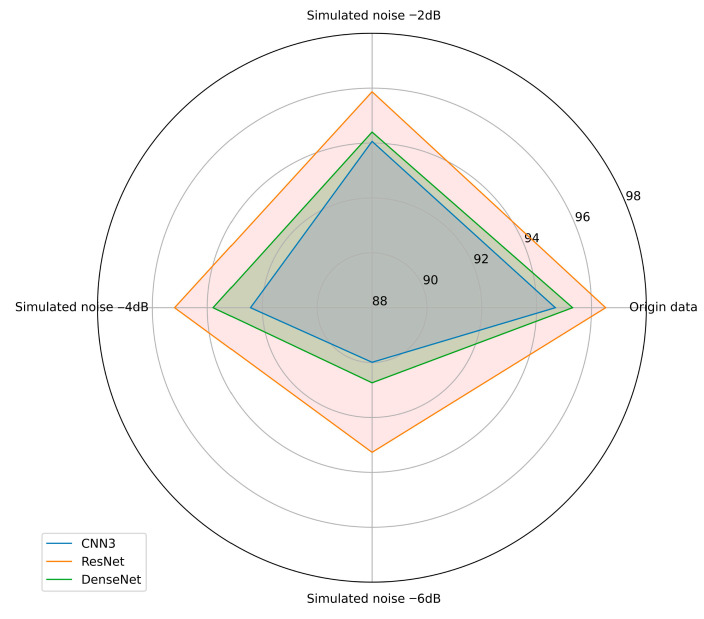
Comparison of model accuracy under different simulated noise levels.

To verify the robustness of the models, we conducted systematic experiments on the three improved models. Specifically, we applied simulated noise of different decibels to the test set data to evaluate their noise resistance capabilities. The experimental results are shown in Figure 13 and Table 9. In these experiments, the noise levels increased from 0 dB to 6 dB to observe the models’ performance under different noise levels. The experimental results showed that the accuracy of all three models did not significantly decrease in noise environments below 4 dB. This indicates that they have good robustness under low noise conditions. However, when the noise level increased to 6 dB, the performance of CNN3 and DenseNet significantly declined, while the ResNet model maintained high accuracy even at 6 dB noise, demonstrating the best noise resistance capability. These results provide important information for assessing the reliability of the improved models in practical applications. It is worth noting that to ensure the fairness of the experiment, we added the same decibels of noise to all three sensors. In actual conditions, the current signal is minimally affected by the environment, so the models’ robustness would theoretically be better than the experimental results.

### 3.3. Visualization Discussion

**Figure 14:**
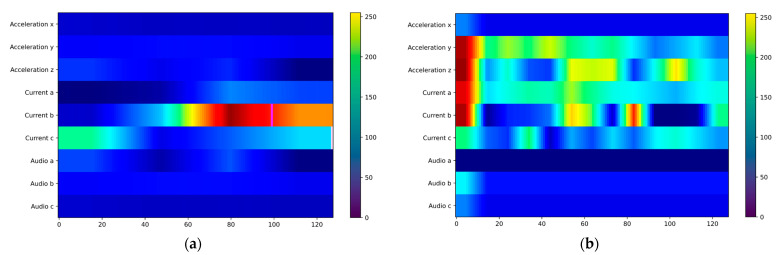

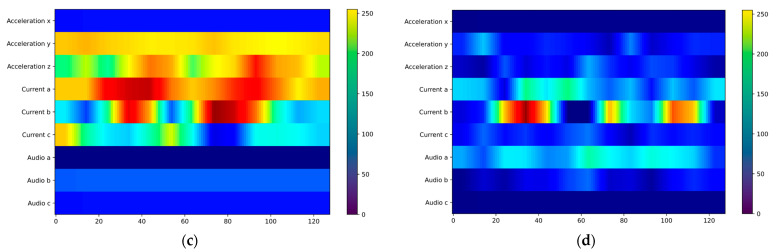
Channel interaction visualization: (**a**) No Load; (**b**) Full Coal; (**c**) Coal Rock; (**d**) Full Rock.

We used Grad-CAM [19] for visualization to more intuitively perceive the feature-capturing ability of the ACI block for multi-sensor data. In the improved CNN3 model, we explored how information interaction enhances feature capturing in each channel. As shown in Figure 14, we extracted heatmaps after inserting the ACI block, with brighter colors indicating more active features and darker colors indicating lower feature weights. Figure 14 shows the activity levels of sensors under various working conditions. The no-load state is the simplest condition, with data from all three sensors showing significant differences from other conditions; the current sensor is most active in Figure 14a. Similarly, the signal differences are pronounced in the all-rock condition; Figure 14d shows that the current and audio sensors are more active. In contrast, the full-coal and coal-rock interface conditions are more difficult to identify than others. At this point, more sensor data is needed for recognition. As shown in Figure 14b,c, multiple channels of the current and acceleration sensors are active, while the audio sensor contributes less to recognition. Using visualization methods, it is demonstrated that the ACI block captures features more comprehensively through information interaction, making fuller use of the multi-sensor data.

### 3.4. Embedded Forecasting Platform

**Figure 15:**
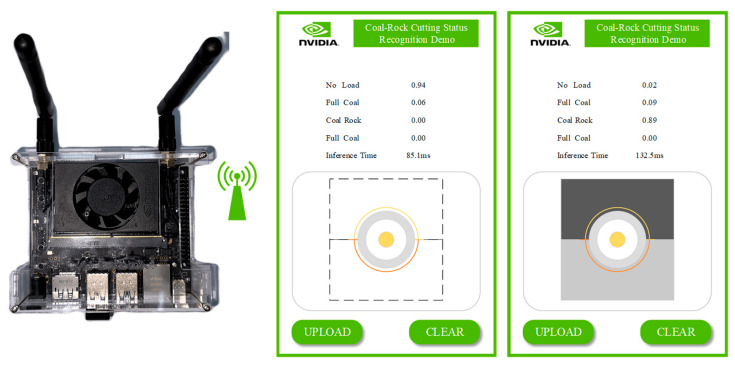
Demonstration of the application running on the NVIDIA Jetson Nano (NVIDIA Corp., Santa Clara, CA, USA).

To evaluate the applicability of the proposed method in resource-limited environments, we tested the actual response speed of the model on an embedded device. The selected device is the NVIDIA Jetson Nano development kit, configured with an ARM A57 CPU and 4 GB of memory. Thanks to the support of NVIDIA JetPack on this device, the deployment and operation of PyTorch models are more efficient. The device and development interface are shown in Figure 15. Using a program monitor, we recorded the prediction time for each time window, as shown in Table 10. The results indicate that using the ACI block does not significantly increase time delay compared to the original mainstream models. For example, in the CNN6 model, the prediction time of CNN3 + ACI block is even shorter with almost the same model performance; the best-performing ResNet + ACI block has a prediction time of 193.4 to 216.1 milliseconds per window, also performing excellently in practical use. This shows that the ACI block can maintain efficient prediction performance on embedded devices without significantly increasing computational delay, making it suitable for resource-constrained work environments.

## 4. Conclusions

This paper proposes a novel graph neural network–based module, the full-channel interaction (ACI) block, for real-time multi-sensor detection of coal and rock cutting status. By encoding, compressing, and implementing feature interaction between channels, the ACI block effectively reduces channel redundancy and enhances feature representation. Experiments on vibration, current, and audio data collected from an experimental platform demonstrate that the ACI block improves the accuracy of mainstream neural networks while maintaining computational efficiency and exhibits strong robustness under noisy conditions. Furthermore, embedded deployment on the NVIDIA Jetson Nano verifies its real-time performance without significant latency increase. Overall, the proposed ACI block enhances model recognition capability, robustness, and applicability in practical scenarios, providing a promising foundation for adaptive online learning, multimodal sensor fusion, and optimized edge computing in future research.

## Figures and Tables

**Table 1:** Experimental platform performance parameters.

Item	Parameters
Cutting motor speed	67 r/min
Drive motor speed	0–90 r/min
Effective cutting diameter	120 mm
Effective itinerary	900 mm
Acceleration acquisition frequency	800 (Hz)
Current acquisition frequency	50 (Hz)
Audio acquisition frequency	800 (Hz)

**Table 2:** Proportion of coal and rock specimens.

Test Piece	Full Coal	Full Rock
Material proportion	4:1	3.5:1
Granulated coal/sand: cement		

**Table 3:** Description of datasets.

Attribute	Sampling Rates	Number of Categories	Total Samples	Proportion of Training Data	Proportion of Testing Data	Sliding Window Size	Test Samples per Class	Sliding Window Step	Sampling Duration per Window	Overlap Rates
Value	50 Hz	4	8000	80%	20%	128	400	64	2.56 s	50%

**Table 4:** Architecture of the CNN3, ResNet, and DenseNet.

	Description	Layer	Kernel Size	Stride	Padding	Output Channels	Activation	Normalization
Model								
CNN3		Layer1	3 × 3	1	1	C (32)	ReLU	×
		Layer2	3 × 3	1	1	C (64)	ReLU	×
		Layer3	3 × 3	1	1	C (128)	ReLU	×
		FC	-	-	-	4	-	-
		Softmax	-	-	-	-	-	-
ResNet		Layer1	7 × 7	2	3	C (64)	-	BatchNorm2d
		Layer2	3 × 3	1	1	C (64)	ReLU	BatchNorm2d
		Layer3	3 × 3	2	1	C (128)	ReLU	BatchNorm2d
		Layer4	3 × 3	1	1	C (128)	ReLU	BatchNorm2d
		Layer5	3 × 3	2	1	C (256)	ReLU	BatchNorm2d
		Layer6	3 × 3	1	1	C (256)	ReLU	BatchNorm2d
		FC	-	-	-	4	-	-
		Softmax	-	-	-	-	-	-
DenseNet		Layer1	7 × 7	2	3	C (64)	ReLU	BatchNorm2d
		Layer2	3 × 3	1	1	C (32)	ReLU	BatchNorm2d
		Layer3	3 × 3	1	1	C (32)	ReLU	BatchNorm2d
		Layer4	3 × 3	1	1	C (32)	ReLU	BatchNorm2d
		Layer5	3 × 3	1	1	C (32)	ReLU	BatchNorm2d
		Layer6	3 × 3	1	1	C (32)	ReLU	BatchNorm2d
		FC	-	-	-	4	-	-
		Softmax	-	-	-	-	-	-

**Table 5:** Model performance indicators.

Model + Method	Accuracy	Recall	Macro F1 Score	Weighted F1 Score	Params
CNN3	92.39%	88.75%	90.45%	90.62%	0.29 M
CNN6	94.33%	91.33%	92.78%	92.95%	0.76 M
CNN3 + SE Block	93.72%	90.86%	92.30%	92.47%	0.29 M
CNN3 + CA Block	94.15%	92.05%	93.08%	93.25%	0.29 M
CNN3 + ACI Block	94.68% (+2.29%)	93.20% (+4.45%)	93.92% (+3.14%)	94.10% (+3.48%)	0.29 M
ResNet	93.85%	91.28%	92.54%	92.72%	0.82 M
ResNet + SE Block	94.52%	92.15%	93.31%	93.49%	0.84 M
ResNet + CA Block	95.36%	93.47%	94.81%	94.68%	0.84 M
ResNet + ACI Block	96.47% (+2.62%)	94.72% (+3.44%)	95.58% (+3.04%)	95.76% (+3.04%)	0.84 M
DenseNet	93.90%	92.52%	93.20%	93.38%	0.57 M
DenseNet + SE Block	94.25%	93.10%	93.66%	93.84%	0.58 M
DenseNet + CA Block	94.76%	93.81%	94.07%	94.28%	0.58 M
DenseNet + ACI Block	95.31% (+1.41%)	94.05% (+1.53%)	94.67% (+1.47%)	94.85% (+1.47%)	0.58 M

**Table 6:** Effect of ACI block inserts in different positions.

After layEr1/Block1	After Layer2/Block2	After Layer3/Block3	CNN3	ResNet	DenseNet
√	-	-	92.94%	94.47%	-
-	√	-	94.68%	95.11%	94.69%
-	-	√	92.94%	96.47%	95.31%
√	√	-	92.26%	96.06%	-
√	-	√	93.41%	94.07%	-
-	√	√	93.14%	94.38%	94.42%
√	√	√	93.64%	94.69%	-

Note: “√” indicates that the ACI block is inserted after the corresponding layer or block; “-” means it is not inserted.

**Table 7:** Comparison of ResNet performance improvement by different parts of the module.

En/Decoding	Massage Passing	ResNet
√	-	95.12%
-	√	95.97%
√	√	96.47%

Note: “√” denotes the component is used, and “-” means it is not.

**Table 8:** Performance comparison of audio channels under different channels.

Input	Accuracy	Recall	Macro F1 Score	Weighted F1 Score
Audio expanded to 3 channels	96.47%	94.72%	95.58%	95.76%
Audio as single-channel	94.80%	93.05%	93.91%	94.10%
No audio data	91.20%	89.50%	90.32%	90.55%

**Table 9:** Recognize the accuracy of each model under different SNRs. (%).

The Noise Type	SNR	CNN3	ResNet	DenseNet
Origin data		94.681%	96.517%	95.312%
Simulated noise	−2 dB	94.061%	95.869%	94.401%
Simulated noise	−4 dB	92.432%	95.190%	93.790%
Simulated noise	−6 dB	89.989%	93.270%	90.736%

**Table 10:** Demonstrate the speed of inference for operations.

Model	Actual Inference Time (Windows/ms)
CNN3	83.7~89.2
CNN3 + ACI Block	91.3~105.4
CNN6	156.7~179.2
ResNet	182.9~210.7
ResNet + ACI Block	193.4~216.1
DenseNet	137.9~158.8
DenseNet + ACI Block	144.2~163.4
Model	Actual inference time(windows/ms)

## Data Availability

The original contributions presented in this study are included in the article. Further inquiries can be directed to the corresponding author.
